# Immunological facets of prostate cancer and the potential of immune checkpoint inhibition in disease management

**DOI:** 10.7150/thno.100555

**Published:** 2024-10-21

**Authors:** Stian Bakke Hansen, Bilal Unal, Omer Faruk Kuzu, Fahri Saatcioglu

**Affiliations:** 1 Department of Biosciences, University of Oslo, Oslo, Norway.; 2 Institute for Cancer Genetics and Informatics, Oslo University Hospital, Oslo, Norway.

**Keywords:** Cold tumor, Combination therapy, Immune checkpoint inhibitor, Immunotherapy, Prostate cancer, Tumor microenvironment.

## Abstract

Prostate cancer (PCa) is the most common non-cutaneous cancer in men and a major cause of cancer-related deaths. Whereas localized PCa can be cured by surgery and radiotherapy, metastatic disease can be treated, but is not curable. Inhibition of androgen signaling remains the main therapeutic intervention for treatment of metastatic PCa, in addition to chemotherapy, radionuclide therapy and emerging targeted therapies. Although initial responses are favorable, resistance to these therapies invariably arise with development of castration resistant PCa (CRPC) and lethal phenotypes. Recent findings have implicated the crosstalk between PCa cells and the tumor microenvironment (TME) as a key factor for disease progression and metastasis, and the immune system is becoming an increasingly attractive target for therapy. Given the striking success of immune checkpoint inhibitors (ICIs) in various cancer types, preclinical and clinical studies have begun to explore their potential in PCa. It has become clear that the PCa TME is largely immunosuppressive, and ICI therapy does not have efficacy for PCa. Intense effort is therefore being made in the field to understand the mechanisms of suppression and to turn the immunosuppressive TME into an immune active one that would enable ICI efficacy. Herein we examine this recent body of knowledge and how the mutational landscape of PCa integrates with an immunosuppressive TME to circumvent ICI-mediated T-cell activity and tumor killing. We then review the emerging potential success of combinatorial ICI approaches, utility of careful patient selection, and potential novel strategies to improve the efficacy of ICI for PCa therapy.

## Introduction

Prostate cancer (PCa) is the most common non-cutaneous cancer in men and a leading cause of cancer-related deaths in developed countries 1. More than 1.4 million new cases and ~375,000 deaths worldwide were ascribed to PCa in 2020, with an expected increase of 17% and 19%, respectively, by 2025 2. Most patients are diagnosed at an early stage for which the mainstay treatment is surgery and radiotherapy, but the risk of potential overtreatment, along with the inherent dangers of treatment-related disease progression, has made active surveillance one common option from a quality of life perspective 3, 4. Despite successful treatment in most patients, PCa recurs in 20-40% of cases within 10 years after the treatment 4, 5. When recurrence occurs, it may be amenable to salvage radiotherapy and androgen-deprivation therapy (ADT), which can be achieved through chemical or surgical castration. However, a significant proportion of patients will eventually relapse into castration-resistant PCa (CRPC) to which any further treatment is rather palliative. Disease management at this point involves novel hormonal therapy (NHT) with antiandrogens or the androgen synthesis inhibitor abiraterone, concurrent with or followed by chemotherapy. Bone metastatic disease is eligible for systemic Radium-233 treatment, and mCRPC patients that present with specific biomarkers may be assigned to receive the novel ^177^Lu-PSMA-617 radioligand therapy or poly (ADP-ribose) polymerase (PARP)-inhibitors 4, 6. Resistance and emergence of advanced disease invariably ensues, such as neuroendocrine PCa and double negative PCa, and new strategies to overcome this are urgently needed.

Immunotherapy is a treatment modality that aims to manage disease by targeting and activating the immune system. In cancer, this involves priming the patient's lymphocytes for destroying the growing tumor rather than attacking features within the cancer cells that are prone to mutation and diversion via bypass pathways. Immune checkpoint inhibitors (ICIs) offer one such strategy and are based on the observation that tumor-infiltrating T cells are often dysfunctional characterized by low activity and expression of co-inhibitory surface receptors 7. These checkpoints serve to maintain self-tolerance in normal physiology, but are subverted in favor of the cancer cell by a complex interplay of chronic inflammation and the cytokine milieu within the tumor microenvironment (TME). Checkpoints include cytotoxic T-lymphocyte associated protein 4 (CTLA-4) and programmed cell death protein 1 (PD-1), which have been studied extensively. Novel targets are emerging, such as the T-cell immunoglobulin and mucin domain 3 (TIM-3), lymphocyte activation gene-3 (LAG-3), and V-domain Ig suppressor of T cell activation (VISTA), that are being investigated for translational applications. The ICI approach aims to release the brakes on the immune system by blocking such co-inhibitory molecules, and a number of monoclonal antibodies are now in clinical use since the first approval of an anti-CTLA-4 agent for advanced melanoma in 2011 8. The resounding success of these checkpoint blockers in melanoma and some other cancers sparked hope for their benefits in PCa 9-11; unfortunately, this expectation has yet to be fulfilled.

The main reason appears to be that PCa has characteristics of a “cold” tumor type with low T-cell infiltrate and intensive immunosuppressive mechanisms. Success of immunotherapy for other cancer types has therefore been difficult to realize for PCa; at present, a dendritic cell-based vaccine (Sipuleucel-T, or Provenge) is the only immune-based treatment specifically approved for PCa 12. Two PD-1 blocking agents have received tissue-agnostic approval based on DNA repair status or mutational burden since 2017 13, yet their performance in PCa is at best questionable, and attempts at targeting other checkpoint molecules have similarly failed in performing under recent data from clinical trials despite showing promise in early stages. A greater understanding of the immunological features of PCa in disease progression is therefore necessary to reactivate the immune compartment in the TME and thereby enhance the clinical benefit of ICIs. Herein we discuss previous setbacks in ICI monotherapies for PCa and how the mutational landscape of PCa integrates with an immunosuppressive TME to circumvent ICI-mediated T-cell activity and tumor killing. We then focus on the emerging benefit of combinatorial approaches, utility of biomarker-informed patient selection, and potential novel strategies to improve the efficacy of ICIs in PCa.

## Tumor heterogeneity is an important hurdle for prostate cancer treatment

Despite many breakthroughs that have emerged in cancer research over the years, the development of treatment resistance and relapse into an aggressive form with poor prognosis remains a major challenge in the clinic. This can mainly be attributed to the heterogeneity of tumors and the complexities of the TME that serve to fuel growth and evolution 14. In essence, the presence of substantial differences at the cellular and molecular levels between patients suffering from a specific subtype of cancer (inter-patient heterogeneity) has been an important driving force for the design of personalized treatment options, the idea of precision medicine. However, the idea of screening a patient's genotype and matching it with a tailored treatment option falters when the heterogeneity extends within a single patient, especially later in the disease course. For example, the striking heterogeneity between PCa foci gives rise to a clear variability in classification, risk stratification, predicted recurrence, and estimated androgen receptor (AR) pathway activity for each patient 15. These findings emphasize the problem of making a clinical decision based on a single lesion that may not be the origin for the lethal stages of PCa.

The immune landscape in cancer is a complex spectrum with many aspects concerning the quantity and characteristics of immune infiltrates; some simplification can be made by assigning positions on each extreme of the spectrum. Neoplasms may be classified by their immunological phenotype into either “hot” or “cold” tumors, wherein the former is recognized by high mutational burden, extensive T-cell infiltration, and markers of both T-cell mediated killing and exhaustion 16. Due to the nature of repeated T-cell stimulation, such tumors are more likely to respond to ICIs. Cold tumors, on the other hand, are characterized by little or no T-cell infiltration, where the cells are restricted to the margins of the tumor (immune-excluded) or missing altogether (immune-desert). Lack of responsive lymphocytes would portend inefficacy of checkpoint blockers, which in fact is what is observed in the clinic. Melanomas, non-small cell lung cancer (NSCLC), and liver cancer, are considered immunologically hot tumors, whereas cancers such as PCa, breast cancer, and pancreatic cancer are recognized as immunologically cold 17-19. Distinction between these immunophenotypes is highly context dependent; all cancer types can present on both sides of the spectrum and thus understanding the mechanisms involved in establishing a cold TME becomes more important than assigning a cancer to any category. This basic understanding is becoming paramount to crack the code that could dramatically improve the success of ICIs for cancer in general, as well as for PCa.

## Immune checkpoint inhibitors are largely ineffective in PCa

In line with the immunophenotype of PCa, ICI therapy has not yielded any significant response in patients with localized or advanced forms of the disease, because there is little release in the TME. Despite showing favorable prostate-specific antigen (PSA) response rates and increases in progression-free survival (PFS) compared to placebo control, the anti-CTLA-4 treatment ipilimumab failed to improve overall survival (OS) in chemotherapy-naïve as well as in patients previously treated with docetaxel and radiotherapy in two separate Phase III studies 20, 21. Severe treatment-related complications were observed in a number of patients that could not justify the benefits of the treatment. Long-term follow-up for the post-chemotherapy study did however find an increase of survivors that becomes pronounced from three years onward 22. In addition, a small Phase II study found an expansion of T cells of effector memory and Th1-like subtypes in patients treated with ipilimumab plus ADT 23, indicating that the ICI evokes a response in a subset of patients the basis of which is not entirely understood. Anti-PD-1 therapy with nivolumab showed tolerable safety profiles in a Phase I study published in 2010 and another from 2012, yet their results demonstrate little activity in PCa 24, 25.

Pembrolizumab is another PD-1 targeting antibody that has found a firm standing in the clinic since its approval for advanced melanoma in 2014 8. Its efficacy, coupled with good safety profiles in other cancer types raised hopes for its successful use in PCa, but it has so far failed to meet expectations. Efforts to identify predictive biomarkers are still ongoing. Intuitively, the expression of programmed death-ligand 1 (PD-L1) in tumor tissue would present a rational marker for stratification of cancer patients for response to anti-PD-1 therapy. With this in mind, a large study published in 2018 observed a significant increase in PD-L1 immunoreactivity in metastatic CRPC (mCRPC) compared to primary PCa 26, where expression is rare, and the Phase II KEYNOTE-199 study took this rationale into practice for mCRPC patients 27. Patients with a treatment history involving docetaxel were enrolled in PD-L1 positive or negative cohorts for administration of 200 mg pembrolizumab every 3 weeks until specified endpoints were reached. Objective response rates (ORR) were equivalent in the two cohorts, with a 5% response in the PD-L1 positive cohort, only 2% higher than the PD-L1 negative group. With a durable response or stable disease in 10% of the participants, it is clear that a small number of mCRPC patients respond favorably to single treatment, but the characteristics of this subpopulation can clearly not be defined by PD-L1 status alone.

In June 2020, the FDA approved the use of pembrolizumab as monotherapy for treatment of advanced solid tumors with a high-mutational burden (TMB), defined by a score of 10 mutations per megabase or higher, that have progressed on prior treatment and for which no effective alternatives are available 28. Mutational burden is often predictive of responses to ICIs, because mutations produce neoantigens that may be targeted by effectors of the immune system and thereby recruit lymphocytes to the tumor. The FDA-approval was considered in the wake of the advancing Phase II KEYNOTE-158 trial (ClinicalTrials.gov: NCT02628067) and a retrospective large-scale whole-exome sequencing analysis, whose 175 mutations/exome criterion is judged equivalent to the established 10 mut/Mb setpoint 28, 29. Consistent with KEYNOTE-158 29, patients with a high mutational burden demonstrated greater responses to the treatment than their counterparts 28.

This is also the case for PCa-patients; however, yet again the overall response rate is very low, peaking at 9% versus 6% in the low-TMB cohort, consistent with a study in ipilimumab-treated patients which suggested that TMB is not a singular decisive factor for ICI responses in PCa 30. The retrospective analysis also suffered from an underrepresentation of PCa cases, with a modest 11 pembrolizumab-treated tissue samples eligible for whole-exome sequencing. A study followed up on this and suggests that pembrolizumab may still be a viable option instead of chemotherapy in mCRPC when the mutational burden is high 31, but additional markers need to be evaluated to maximize treatment benefits against the cost. Note that mismatch repair deficiencies have been suggested reasonable predictors across tumor types 32, and the responses within mismatch repair deficient cohorts appear to be beneficial in PCa 33, 34 although the studies in question are limited by sample size.

## The prostate cancer immunophenotype is characterized by low mutational burden and active suppression

### The mutational landscape of PCa

The mutational landscape of PCa is multidimensional but rather sparse. Overall, patients present with a low mutation frequency, covering less than 1 mutation per megabase (mut/Mb) in primary disease and up to an average of 4 mut/Mb for advanced metastatic disease, albeit with significant variation between patients 35, 36. Hypermutated phenotypes with higher frequencies do associate with tumors that have mismatch repair deficiencies (commonly dMMR), most often due to alterations in *MSH2*, *MSH6* and *MLH1* genes, yet these make up only a small subset of cancer patients 37. PCa is therefore significantly different from the archetypical hot cancers such as melanoma and lung cancer that present with a median frequency close to 10 mut/Mb and much higher maximal representation 38. Instead of a high mutational burden, structural lesions at the chromosomal level are widely recognized in PCa with fusion events being most common and often causing overexpression of oncogenes in the E26 transformation-specific (ETS) transcription factor family downstream of androgen-regulated promoters 35, 39. The *TMPRSS2:ERG* fusion alone coincides with around 50% of all PCa cases and makes up 90% of ETS-related fusion events, but other androgen-sensitive fusion partners such as *NDRG1* and *SLC45A3* are also frequently observed 40.

Other large-scale chromosomal events in PCa involve amplification of regions 8q and Xq and more commonly deletions in regions 8p, 10q, 13q, and 17p; these regions encompass genes that are strongly linked to PCa progression, including the *AR* gene, the *MYC* oncogene, and tumor suppressors *PTEN* and *TP53* 35, 41. Coincidental amplification or deletion involving two or more of these has been linked with aggressive cancer traits in patients and preclinical models 42-45. The Cancer Genome Atlas (TCGA) Research Network published a paper in 2015 that delineated the molecular taxonomy of primary PCa that resulted in the classification of primary disease into seven main clusters, which are characterized by gene fusions or single gene mutations 36. According to this classification, ETS-related fusion events and overexpression (*ERG*, *ETV1*, *ETV4*, *FLI1*) constitute 4 of the 7 clusters, the final 3 defined by coding mutations or copy-number variations in the Speckle-type POZ protein (*SPOP*), Forkhead box protein A1 (*FOXA1*), and Isocitrate dehydrogenase 1 (*IDH1*) loci. The dependency on AR signaling in PCa pathology is accentuated by the fact that the proteins encoded by these genes have been associated with AR protein activity, either by physical protein-protein interactions or by being AR target genes 46-49.

Genetic abnormalities give rise to antigens that can be broadly classified as tumor-associated or tumor-specific antigens (TAA and TSA, respectively), depending on their expression pattern in healthy tissues. Several detailed reviews have been published in recent years that incorporate advances in cancer vaccine strategies with a discussion of antigen selection for maximizing benefit in the clinic 50, 51. TAAs are autologous but characterized by abnormal expression in cancer versus benign tissue: overexpressed antigens, cell of origin lineage-specific differentiation antigens, and cancer-testis (CT) antigens. The latter group covers a number of potential targets such as melanoma-associated antigen 1 (MAGE-A1), New York esophageal squamous cell carcinoma 1 (NY-ESO-1), and Kita-Kyushu lung cancer antigen 1 (KK-LC-1), that are normally restricted to germline tissue but overexpressed in cancer 51, 52. PCa is no exception, and CT antigens of the MAGE-A and CSAG protein subfamilies are particularly abundant in advanced PCa 53.

Since the testes constitute an immune privileged site and because germline cells express low to no MHC class I molecules on their surface 51, CT antigens evade interaction with the immune system until their ectopic expression in cancer cells leads to their recognition as foreign. With minimal peripheral tolerance mechanisms at play, such molecules have the potential to elicit strong immune responses despite being self-antigens and have proven strong candidates for cancer vaccines 51. A major challenge is the elevated risk of inducing autoimmunity, and careful vaccine design involving these antigens is paramount to favor benefit above risk. Recently, the epigenetic reader protein Tudor domain containing 1 (TDRD1) was recognized as central to the biogenesis of small nuclear ribonucleoproteins (snRNPs) in PCa 54. The germline protein is ectopically expressed in more than half of clinical cases, and its ablation disrupts the cellular snRNP machinery as well as suppressing proliferation *in vitro*, while also increasing antiandrogen sensitivity. These observations serve as reminders that CT antigens and TAAs in general have inherent value as actionable targets aside from their potential as active vaccine components, adding another layer to their clinical relevance.

TSAs are by definition highly immunogenic because they are recognized as foreign. Such antigens may be derived from viral oncogenes or tumor-specific mutations that generate neoantigens, which are also promising candidates for the active component in therapeutic cancer vaccines 50. Independent studies have observed increased survival and potentially favorable responses to ICIs in patients with higher estimated neoantigen load and T-cell activation signature in different cancer types 55-57. Furthermore, recent publications suggest that neoantigen-specific B-cells and CD4^+^ helper T cells, in particular T follicular helper cell subsets, can strongly promote antitumor immunity by enhancing effector cell function, sparking the possibility for synergy between neoantigen vaccines and ICI treatment 58, 59. Because its mutational landscape favors a low neoantigen burden, however, the typical PCa presents with a panel of antigens that by themselves may not be optimal inducers of the immune response; furthermore, without a population of high-quality neoantigens on display for effector cells, the tumor can more easily escape immune recognition and destruction. As such, the immunogenicity of mutated gene products, or rather lack thereof, would discourage mass B- and T-cell infiltration and render the PCa tumor immunologically cold.

### Characteristics of an immunosuppressive microenvironment

**Figure 1:**
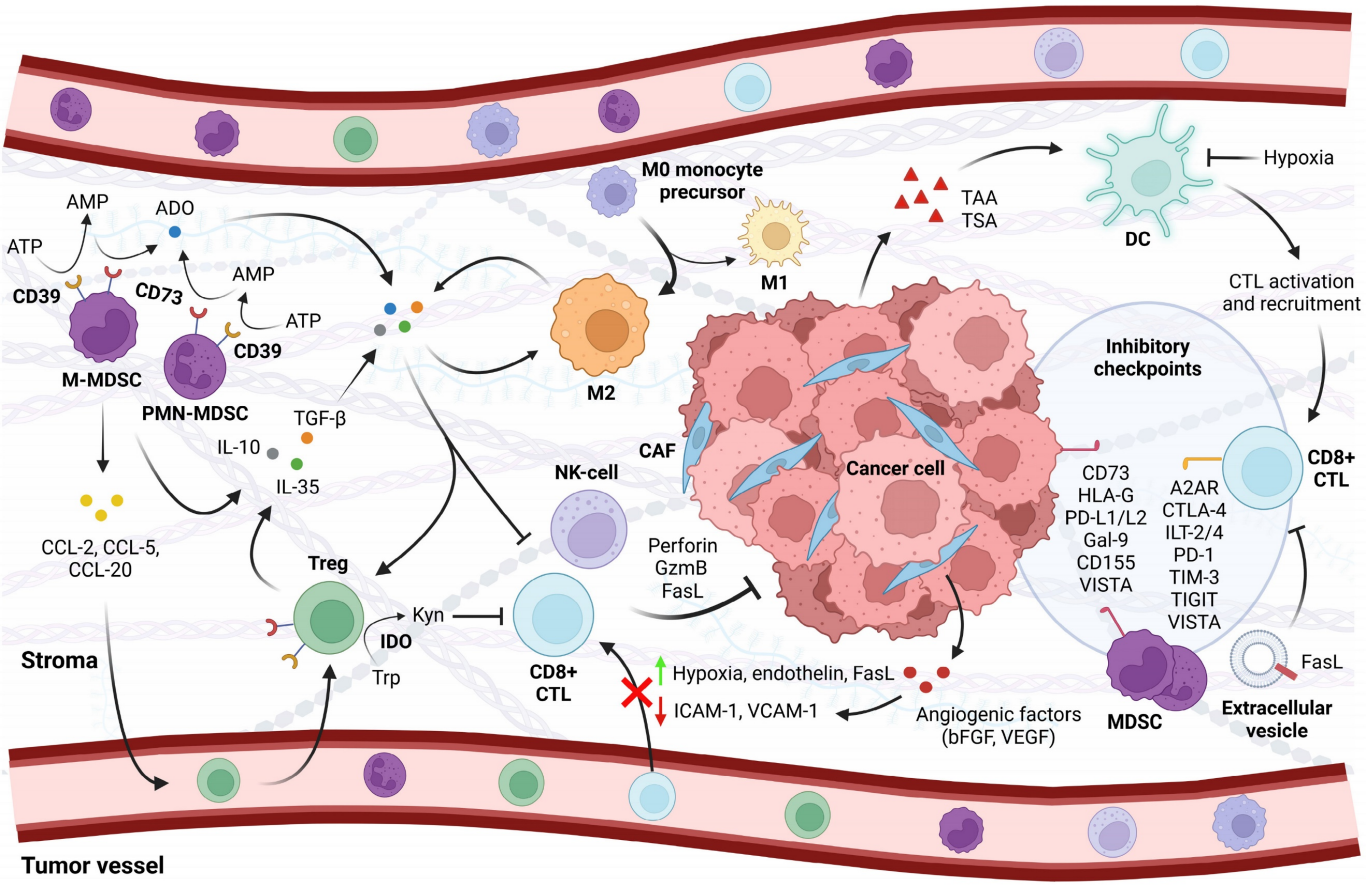
** Characteristics of the PCa TME**. Antigen recognition by dendritic cells leads to the activation and recruitment of CD8^+^ cytotoxic T lymphocytes (CTLs) to the tumor. A modified cytokine milieu discourages CTL recruitment and favors the infiltration of CD4^+^ regulatory T cells (Tregs), pro-tumorigenic M2-polarized macrophages, and myeloid-derived suppressor cells (MDSCs) that cooperate in maintaining an immunosuppressive environment. Exposure to angiogenic factors from cancer cells and surrounding fibroblasts depletes adhesion molecules in the endothelial lining that are required for CTLs to extravasate; furthermore, cells that successfully infiltrate the stroma are subject to inhibition by hypoxia, local metabolite depletion, and pro-tumorigenic cytokines. Upregulation of Fas ligand in the vasculature and on tumor-derived exosomes initiates apoptosis in CTLs without affecting cells with stronger apoptotic defenses. As a final barrier to antitumor immunity, cancer cells and the immunosuppressive cell types engage inhibitory checkpoints that are currently attractive targets in the clinic. Also shown are natural killer (NK) cells, which are similar to CTLs in their mode of action and subject to similar constraints. A2AR = adenosine A2A receptor, Ado = adenosine, AMP = adenosine monophosphate, ATP = adenosine trisphosphate, bFGF = basic fibroblast growth factor, CAF = cancer-associated fibroblast, CCL-2/-5/-20 = chemokine ligand-2/-5/-20, CD73/-155 = cluster of differentiation 73/-155, CTL = cytotoxic T lymphocyte, CTLA-4 = cytotoxic T-lymphocyte-associated protein 4, DC = dendritic cell, FasL = Fas ligand, Gal-9 = galectin 9, GzmB = granzyme B, HLA-G = human leukocyte antigen G, ICAM-1 = intercellular adhesion molecule 1, IDO = indoleamine 2,3-dioxygenase, IL-10/-35 = interleukin-10/-35, ILT-2/-4 = inhibitory receptors Ig-like transcript 2/-4, Kyn = kynurenine, MDSC = myeloid-derived suppressor cell (M = monocytic, PMN = polymorphonuclear), NK-cell = natural killer cell, PD-1 = programmed death protein 1, PD-L1/-2 = programmed death-ligand 1/-2, TAA = tumor-associated antigen, TGF-β = transforming growth factor β, TIGIT = T-cell immunoreceptor with immunoglobulin and immunoreceptor tyrosine-based inhibitory motif domains, TIM-3 = T cell immunoglobulin and mucin domain-containing protein 3, Treg = regulatory T cell, Trp = tryptophan, TSA = tumor-specific antigen, VCAM-1 = vascular cell adhesion molecule 1, VEGF = vascular endothelial growth factor, VISTA = V-domain Ig suppressor of T cell activation.

In addition to mutational burden, the immunophenotype of a cancer is subject to active immune suppression through physical cell-to-cell contact and chemical modulation. Indeed, earlier studies in murine models demonstrated that primary tumors quickly establish an immunosuppressive environment, gradually impairing the immune system in its ability to act upon insults over time (e.g. 60). Such restraints may occur at any point during recruitment and trafficking up to effector cell action, resulting in a population of inactive cells or a scarcity of immune cells altogether. The immune landscape in PCa is generally sparse in tumor-reactive effectors, although it shows significant heterogeneity in infiltration that may yet show prognostic value 61, 62. The immune cell composition of PCa encompasses a number of cell types including CD8^+^ cytotoxic T-lymphocytes (CTLs) and B-cells, natural killer (NK) cells, and neutrophils, as well as M1-polarized macrophages that exert pro-inflammatory activities 62, 63. Importantly, however, PCa tumors and peripheral blood in patients are enriched with cellular subtypes with immunosuppressive gene signatures: anti-inflammatory M2-polarized macrophages, regulatory T cells (Tregs), and myeloid-derived suppressor cells (MDSC) of monocytic or granulocytic origin 64, 65. These immunosuppressive cell types function by mobilizing immune checkpoints and by secreting immunosuppressive cytokines such as interleukin (IL)-10, IL-35, and transforming growth factor ꞵ (TGF-ꞵ) that inhibit effector cell maturation or function (**Figure 1**) 66-68.

A recent single-cell RNA-Seq study on primary PCa identified a deficit in T-cell cytotoxicity score compared with a group of hot tumors, along with an increase in T-cell exhaustion markers relative to the healthy prostate 63. This coincided with an increased Treg activity score that correlated with a monocytic MDSC-like signature and reciprocal expression of chemokine receptor CCR6 with its cognate ligand CCL20, suggesting that they cooperate to maintain a suppressive TME. Although this particular study did not explicitly evaluate of CD8^+^ T cell density, another study found that the median density for PCa patients (51 cells/mm^2^) is dramatically lower than reported in its melanoma counterpart (approx. 2500 cells/mm^2^ in anti-PD-1 responders) 69-71.

In contrast, a paper published in 2020 addressed the CD8^+^ infiltration in 84 different tumor types and detected a much smaller difference in median density between PCa and melanoma, yet the range was greater for melanoma which peaked at a maximum of about four times higher than PCa with its 499 cells/mm^2^ 72. Despite the limitation of small sample sizes, such data demonstrate that the cold characteristics of PCa are manifested in a functional loss together with quantitative deficiency of CTLs; the prognostic value of intraprostatic CD8^+^ T cells is therefore actively being investigated. This is a rather complex task, however, as some studies associate high numbers of CTLs with increased time to biochemical recurrence and overall survival after surgery 70, 73, while others find a shorter time to cancer progression with high cell density 74, 75, suggesting a context dependency the basis of which is currently unclear.

In one of these studies, the negative prognosis for CD8^+^ cell density was dependent on high expression in the adjacent epithelium of CD73, an immune checkpoint that acts upon the adenosinergic pathway to suppress antitumor responses (**Figure 1**) 75. Coincident with low CD73, the association between CD8^+^ density and time to recurrence was therefore not significant. These findings suggested that CD73 may mechanistically aid the conversion of CTLs to non-conventional immunosuppressive CD8^+^CD25^+^ subtypes, which have previously been described in PCa 75, 76. Similar to CD4^+^ Tregs, these subpopulations are capable of suppressing effector T-cell function by contact-dependent and -independent mechanisms; however, the mechanisms are not completely elucidated and current evidence challenges the relevance of IL-10 and TGF-β that are well characterized products of conventional Tregs 76, 77. It is therefore important to recognize that the CD8^+^ T-cell compartment is more dynamic when evaluating its clinical implications. Current data are more consistent with regards to the prognostic value of Tregs 78 and MDSCs 79, whose overrepresentation is associated with worse prognosis for PCa patients.

Activated T cells upregulate receptors including CXCR3 and CCR5 to recognize chemokine ligands in the TME, which then aid their functional maturation and navigation into the site of insult 80. Elimination of these cytokines by proteolytic cleavage 81, transcriptional repression 82, or epigenetic silencing 83, 84 is a means by which tumors can impair infiltration; by precise fine-tuning of the cytokine milieu the PCa cells tip the scales in favor of immunosuppressive rather than antitumor effector cells 85-87. Ectopic expression of components in the Polycomb repressive complex 1 (PRC1) promoted self-renewal and metastasis in double-negative mCRPC (DNPC), while concurrently recruiting Tregs, tumor associated macrophages (TAMs), and MDSCs, mainly by production of the cytokine CCL2 88. Inhibition of PRC1 suppressed recruitment of immunoinhibitory cell types and dramatically increased the efficacy of double checkpoint immunotherapy (anti-CTLA-4 + anti-PD-1) in murine models for DNPC, indicated by the significant decline in tumor burden as well as emergence of CTLs and CD4^+^ effectors 88. These findings suggest that PRC1 could serve as a rational target for PCa, and also underscores the potential for combination therapies to improve clinical outcomes with ICIs in PCa, as discussed below.

Infiltration of immune cells into the TME can be hindered at the endothelial level by modifying expression of adhesion proteins such as intercellular adhesion molecule 1 (ICAM-1) and vascular cell adhesion molecule 1 (VCAM-1), which are necessary for leukocyte trafficking and extravasation 89. Consistently, prolonged exposure to angiogenic factors such as vascular endothelial growth factor (VEGF) and basic fibroblast growth factor (bFGF) effectively reduced the inflammatory upregulation of these molecules in the endothelial lining, thereby preventing T-cell adhesion and transendothelial migration (**Figure 1**) 90; other studies suggested that angiogenic factors cause clustering defects with similar outcome 91. This effect has been denoted *tumor endothelial cell anergy* due to the emerging insensitivity to inflammatory cytokines. Anti-angiogenic treatment counters this suppression and enhances infiltration 92. The angiogenic signature is suggested to be prognostic in PCa and suggests potential targets of combinational benefit 63, 93.

Furthermore, the endothelial compartment appears to have a profound selectivity between infiltrating immune cells. The common lymphatic endothelial and vascular endothelial receptor (CLEVER-1) is upregulated in hepatocellular carcinoma with a preference for Tregs more than other T-cell subsets 94. Endothelin B receptor overexpression has been associated with lower CTL recruitment but larger Treg populations in gliomas 95, while a receptor antagonist increases overall T-cell infiltration in preclinical models of ovarian cancer 96. There is sparse knowledge on the immunological impact of vascular endothelin receptors in PCa, but endothelin ligands are increased in patients and found to aid in tumor growth and metastasis 97, suggesting that a similar mechanism may be involved. Interestingly, tumor-derived cytokines have been found to upregulate Fas ligand in the surrounding vasculature of tumors including PCa, selectively engaging the extrinsic pathway of apoptosis in CD8^+^ T cells but not in Tregs, which have stronger anti-apoptotic barriers (**Figure 1**) 98. Studies on PCa cell lines have also reported the release of Fas ligand in soluble form 99 or as part of tumor-derived exosomes that induce cell death in CD8^+^ lymphocytes 100.

Immune cells that successfully extravasate are rendered inactive by a hostile microenvironment. The tumor vasculature is abnormal with an endothelial lining that is loosely connected, highly irregular, and under pressure that causes individual vessels to collapse 101. The result is an oxygen deficit accompanied by acidosis which inhibits functional CTL maturation 102. Activation of hypoxia-inducible factor 1 alpha (HIF-1ɑ) engages a transcriptional program that impairs dendritic cell activation and CTL function 103, 104, and leads to recruitment and activation of Tregs and MDSCs as well as polarization of immunosuppressive M2 macrophages in a number of cancer types 105-107. Such hypoxic zones are prevalent in clinical and preclinical PCa and behave as immune-privileged sites that may be targeted for therapeutic benefit 108. Administration of the hypoxia-activated prodrug evofosfamide leads to a collapse of these areas in the TRAMP-C2 PCa mouse model and improves T-cell infiltration at the expense of immunosuppressive cell types 108. Hypoxia reduction sensitizes the tumors to CTLA-4/PD-1 checkpoint blockade as demonstrated by a robust increase in CD8^+^ T-cell effector function and tumor rejection.

Protumorigenic cytokine profile and hypoxia stimulate immune checkpoints including CTLA-4, TIM-3, lymphocyte activation gene-3, and VISTA on T cells or other components of the TME 109, 110, which may directly inhibit CTL function 109, 111, 112 or stimulate suppressor cell activity (**Figure 1**) 110. PD-1 has been identified on the surface of PCa-infiltrating CD8^+^ T cells 113, its ligands PD-L1 and PD-L2 are frequently overexpressed in the prostate TME 114, 115; in addition, the compensatory upregulation of VISTA in PCa that is treated with ipilimumab suggests an emergent role of this checkpoint that warrants further investigation 116.

Although current immunotherapies are mainly directed at tumor-specific T cells, an increasing body of evidence points towards innate NK cells as important contributors to tumor immunity, and means of harnessing their potential is a point of interest 117. NK cell infiltration and activity has been associated with PCa stages and prognosis 118, 119, emphasizing their relevance. Similar to their adaptive counterparts, NK cells are subject to immune checkpoint activity; consistently, Pasero *et al.* (2016) observed a shift in surface profile of activating receptors (e.g. NKp46 and NKG2D) that were underrepresented and inhibitory receptors (e.g. ILT2 and NKG2A) were overexpressed in tumor-infiltrating NK cells of PCa patients 119. This coincided with an immature phenotype and impaired NK cell function. This was supported by another study where an altered phenotype in the circulating fraction of NK cells in patients was observed, including NKG2D downregulation and upregulation of exhaustion markers TIM-3 and PD-1, again suggestive of an impaired NK cell state 120. Taken together with reports that PCa cells actively employ mechanisms to evade recognition and NK cell-mediated killing 121, 122, these observations suggest that remobilizing the NK cell compartment to attack the evasive tumor may be a fruitful therapeutic approach.

## Potentiating immune checkpoint inhibitors

### Combining multiple checkpoint inhibitors and other immune-targeting agents

With the fluctuating success of ICI monotherapies, efforts have been ongoing to assess the clinical effects of combinatorial treatment. Following its approval for BRAF V600 wild-type advanced melanoma in 2015, the combination of nivolumab and ipilimumab has expanded its range to other cancer types including hepatocellular carcinoma, advanced renal cell carcinoma, and NSCLC for patient subgroups 8. On several occasions nivolumab plus ipilimumab has been superior to either monotherapy, and it is suggested that combination treatment may achieve synergy due to differential mechanism of action for CTLA-4 and PD-1 blockade that integrate for a unique outcome 123, 124. Taking into account the compensatory upregulation of PD-L1 that has been demonstrated in PCa patients treated with ipilimumab 116, the ongoing CheckMate 650 trial is evaluating whether the dual targeting of PD-1 and CTLA-4 may improve the clinical performance in patients with mCRPC (ClinicalTrials.gov: NCT02985957) 125. Initially, 90 patients were evenly divided into chemotherapy naïve and post-chemotherapy cohorts and treated with 1 mg/kg nivolumab and 3 mg/kg ipilimumab every three weeks for up to four doses, followed by 480 mg nivolumab every four weeks until cancer progression or threshold toxicity. Preliminary data from the trial inspired a carefully optimistic point of view, with a small number of patients achieving complete responses and objective response rates of 25% and 10% for pre-chemotherapy and post-chemotherapy patients, respectively 125.

Although caution must be exercised when comparing studies of different design, the survival benefits for the pre-chemotherapy population was higher than for either treatment alone 125. The median 15.2-month OS is notably better than the 11.2 months for ipilimumab alone 21; with its objective response rate, it also improved on the outcome of nivolumab monotherapy 25. Because of high discontinuation rates during combination dosing or monotherapy maintenance, however, the current Phase II study was expanded to cover dose and schedule modifications. 259 post-chemotherapy patients were randomly assigned 2:2:1:2 to four cohorts, with cohort D1 and D2 receiving a modified combination treatment 126. Patients in D1 received 3 mg/kg nivolumab plus 1 mg/kg ipilimumab every three weeks up to four doses, whereas D2 received 1 mg/kg nivolumab every three weeks up to eight doses and 3 mg/kg ipilimumab every six weeks up to four doses. Both were followed up with 480 mg nivolumab every four weeks.

So far, 15% and 26% of patients have discontinued due to toxicity in the two cohorts, which represents a slight improvement over withdrawal in the first report (36% in the post-chemotherapy cohort) 125, 126. Preliminary analyses report 9% and 15% objective response rate for cohorts D1 and D2, along with a median OS of 15.9 and 13.5 months, respectively. Interestingly, the chemotherapy arm (D4) was associated with lower discontinuation rates and greater PSA responses than each of the combination cohorts 126. 24% of patients with baseline measurable disease achieved a >50% PSA decline in cohort D4, compared to 14% and 18% in cohorts D1 and D2. Given a 14.8 month median OS, this raises a question as to when the trade-off between efficacy and toxicity makes the checkpoint combination more beneficial.

The upregulation of VISTA that emerged in PCa patients treated with ipilimumab raises a question of how it fits in with the timeline in cancer development 116: does it emerge as a backup checkpoint when other checkpoints fail to protect the tumor, or does it have other functions that directly fuel cancer growth and metastasis? Could VISTA be a positive modulator of antitumor immunity that aims to counter a suppressive environment as suggested in other cancers 127? These questions are still open, but a recent study found a significant increase in VISTA-expression on circulating CD8^+^ T cells in PCa patients undergoing stereotactic body radiation therapy (SBRT) 128, further supporting a role for the checkpoint in PCa that warrants further investigation. With this background, a VISTA-targeting antibody, KVA12123, alone or in combination with pembrolizumab, has entered the recruitment stage for a Phase I/II study in patients with refractory or relapsed advanced solid tumors (VISTA-101, NCT05708950). Other immune checkpoints are gaining more interest as potential targets in recent years, including the adenosinergic CD73-axis 75, 129 and the non-classical Human leukocyte antigen (HLA)-G 119, 130, 131, both of which are being investigated in clinical trials for PCa (see **Table 1**).

Advances in technology allow for more precise targeting of anticancer agents for greater therapeutic benefit; particularly the development of bispecific antibodies (bsAbs) is bringing the field of immunotherapy a step forward 132. Unlike the aforementioned antibodies which are designed to bind a specific epitope, bsAbs are engineered with binding sites that have different specificities and thereby coordinate separate functions including blocking 133, drug delivery 134, and T-cell engagement to TAAs 135. T-cell engaging bsAbs targeting prostate-specific membrane antigen (PSMA) 136, delta-like protein 3 (DLL3) 137, and HER2 138 are showing promise in preclinical and early clinical trials for PCa, as is a trispecific PSMA-targeting T-cell engager that is derived from the half-life extended TriTAC platform 139. Even bsAbs targeting costimulatory molecules are demonstrating clinical activity concurrent with checkpoint blockers 140.

Data on double checkpoint targeting bsAbs in PCa are currently sparse, but constructs that simultaneously interfere with the PD-1 and LAG-3 or CTLA-4 axes have demonstrated activity in preclinical models 141; consistently, a PD-1/CTLA-4 bsAb performed well in a Phase I trial that included mCRPC patients 142. 13% of evaluable patients achieved objective responses, including two CRPC patients with confirmed PSA responses. This bsAb called vudalimab (XmAb®20717) has now advanced into two Phase II studies that encompass mCRPC, one of which evaluates the antibody alone or in combination with chemotherapy or olaparib in 5 molecular subtypes (NCT05005728) 143, 144. Similarly another PD-1/CTLA-4 bsAb derivative is in a Phase I study for mCRPC 145. Once the mechanistic role of VISTA in PCa is more fully understood, dual targeting with VISTA/CTLA-4 or VISTA/PD-1 bsAb may also prove beneficial in the clinic.

Because the immunosuppressive TME is more than the sum of its checkpoints, an alternative strategy would be to target both checkpoints and cytokines that protect the tumor, such as TGF-ꞵ, with a single agent 146. Bintrafusp alfa (M7824) is a bifunctional protein that fuses the extracellular domains of the TGF-ꞵ receptor II to the C-terminal end of a humanized anti-PD-L1 heavy chain; it has demonstrated potent activity in preclinical models along with manageable safety profiles in heavily pretreated cancer patients 147, 148. Independent studies have shown that M7824 depletes soluble TGF-ꞵ and increases T-cell trafficking into tumor sites along with antigen-specific CD8^+^ T-cell mediated cytolytic activity, but it also enforces changes in the microenvironment in favor of the immune system to potentially increase the efficacy of cancer vaccines 148, 149. With this rationale, a Phase II study is currently evaluating a triple-attack in patients with biochemically recurrent PCa (NCT03315871). The triple treatment comprises the viral vector based PROSTVAC-V/F regimen 150 in combination with a CV301 prime-boost system of viruses expressing TAAs carcinoembryonic antigen (CEA) and mucin-1 (MUC-1) plus TRICOM [triad of costimulatory molecules (B7.1, ICAM-1, and lymphocyte function-associated antigen 3 (LFA-3)], and bintrafusp alfa. The combination of checkpoint blockers and antitumor vaccination holds significant potential deserving of further research (see **Table 2**) 59, 151.

### Combining checkpoint inhibitors with standard of care treatment and targeted therapy

Conventional chemotherapeutics have been found to promote an immunomodulatory effect that exceeds direct tumor cell cytotoxicity 152. This includes the induction of immunogenic cell death within tumors 152 and direct activation of effector cells 153. Docetaxel, a standard line of treatment for CRPC, was found to promote the differentiation of antitumorigenic M1 macrophages *in vitro* and enhance CD8^+^ T-cell effector function in murine models for colon cancer 154; however, CRPC seems to adapt to docetaxel-induced damage by activating pro-tumorigenic M2 macrophages 155. This is a potential mechanism for chemotherapy resistance in late-stage PCa that constitutes a significant hurdle. Paradoxically, docetaxel-based therapy has proven clinically favorable in remodeling the TME, with paired pre- and post-therapy tumor samples showing statistically significant increases in CD8^+^ T-cell infiltration for patients with locally advanced PCa 153. Taken together, the immunological effects of such treatments have very important implications that have previously been alluded to: Effects of immunotherapy may be improved with established treatment strategies that concurrently remodel the tumor immune landscape. This has been suggested by a study that combined sipuleucel-T with ipilimumab in mCRPC 156 and could be a new paradigm for clinical trials that investigate the combinatorial potential of ICIs, such as the Phase II CheckMate 9KD Trial that investigates the efficacy of nivolumab in combination with either docetaxel 157, the PARP-inhibitor rucaparib 158, or enzalutamide in mCRPC patients (NCT03338790).

Studies have shown that the success of ADT in early metastatic disease is associated with a remodeling of the immune infiltrate that could render the cancer susceptible to ICIs 159, but the window of opportunity to progression and therapy resistance could be extremely narrow. This raises the question as to how checkpoint blockade fits within the treatment timeline for PCa patients, whether early or treatment-naïve patients would achieve more durable responses than late-stage CRPC patients, and trials are ongoing on different ends of the spectrum. As an example, the Phase II PEAPOD_FOS trial is assessing the efficacy of PD-1 blockade in combination with cabazitaxel and carboplatin for patients with aggressive variant mCRPC (NCT05563558), while another Phase II trial is evaluating the combination of PD-1 blockade with ADT and docetaxel in newly metastatic hormone-sensitive PCa (mHSPC, NCT03951831). It is possible that studies would benefit from directly comparing responses at different portions of the treatment spectrum, but patients with mCRPC that have progressed on prior treatments with limited options are more likely to enroll in these studies than patients that have yet to undergo treatment. The PROSTRATEGY trial is assessing whether a double ICI approach could provide significant survival benefit with simultaneous chemotherapy and ADT when introduced to patients with mHSPC (NCT03879122). Other ongoing trials with dual checkpoint inhibition and standard of care treatment modalities are presented in **Table 3**.

Radiotherapy is a mainstay treatment for PCa patients with progressive localized disease and as salvage treatment for biochemically recurrence after radical prostatectomy 4. Due to the mutagenic and cytotoxic nature of targeted radiation, it is capable of mobilizing immune responses against the primary tumor and even tumors away from the irradiation site, an effect that may potentially improve ICI responses in the clinic 160, 161. Efforts are therefore ongoing to investigate the efficacy of radiotherapy in combination with PD-1/PD-L1 blockers, e.g. as first-line treatment with atezolizumab (Tecentriq) and ADT (NCT04262154), and as salvage treatment with pembrolizumab (Pembro-SRT, NCT04931979). Preliminary results from a study combining nivolumab with brachytherapy and external beam radiotherapy (EBRT) in high-grade PCa are encouraging, and have therefore advanced into a Phase II trial (NCT03543189) 162. In addition to targeted radiation, systemic radiotherapy using radioactive isotopes has been used in the clinic for advanced PCa, with the FDA approvals of Radium-233 for bone metastatic CRPC and the radioligand ^177^Lu-PSMA-617 for PSMA-positive mCRPC 6, 163. Combination treatments with these systemic radiotherapies and ICIs are currently being explored, and alternative radiation sources like an Actinium-225 construct targeting the novel PCa marker CD46 may emerge as promising candidates in the future 164.

Notably, the observation that Radium-233 alters the expression pattern of PD-1 in infiltrating CD8^+^ T-cell subsets makes it a candidate for combination therapy 165. However a Phase II study combining Radium-233 with pembrolizumab failed to increase immune infiltration into bone metastases and did not affect the secondary outcomes of OS and PFS 166. Another trial tested a combination of Radium-233 with atezolizumab and found increased toxicity compared to each treatment alone without any survival benefit for mCRPC patients 167. Other trials are ongoing, including a randomized Phase I/II study that combines the PD-L1 blocker avelumab with Radium-233 and M3814, an inhibitor of double-stranded break repair enzymes, with the hypothesis that this may enhance direct tumor killing and immune mobilization (NCT04071236). ^177^Lu-PSMA-617 was approved more recently and is therefore not as extensively evaluated in combination treatments, but it has shown a favorable safety profile and indications of activity in combination with pembrolizumab 168; it is currently being tested with dual checkpoint inhibition in the Phase II EVOLUTION trial (see **Table 3**). *In vivo* studies and a patient case suggest increased efficacy of the radioligand when preceded by EBRT, which could add another layer of control with checkpoint blockers in management of PCa 169.

Along with the traditional strategies introduced above, targeted therapies are becoming increasingly attractive in PCa treatment. Given the central role of AR signaling in the growth and maintenance of PCa, AR itself is emerging as a promising target for ICI combination therapies 170. Two Phase III studies assessed the combination of pembrolizumab with enzalutamide vs placebo and enzalutamide in mCRPC (KEYNOTE-641) and mHSPC (KEYNOTE-991), both of which were recently discontinued due to lack of efficacy 171, 172. A similar trial assessed PD-L1 blockade with atezolizumab and enzalutamide, which also failed to achieve primary endpoints, but exploratory analyses associated better responses with CD8^+^ infiltration, TMB, PD-L1 expression, and phosphatase and tensin homolog (PTEN) loss, all of which have been alluded to previously 173. Rather than discouraging further studies into the combination of NHT and ICIs, these results should encourage efforts into understanding mechanisms of treatment resistance in greater detail and biomarkers that can prospectively predict treatment responses. Such is the rationale for ongoing biomarker-selected studies like the Phase II GUNS study, where patients with hypermutated phenotypes, microsatellite instability, Lynch syndrome or *CDK12* alterations are assigned to a cohort receiving ADT with NHT and PD-L1 blockade (NCT04812366).

The only PCa approved targeted treatment, aside from conventional AR-targeting agents, involves the PARP-inhibitors olaparib and rucaparib. They were approved by the FDA for use in DNA damage repair deficient mCRPC that has progressed on NHT, and rucaparib has docetaxel treatment as a final prerequisite 8. The Phase Ib/II KEYNOTE-365 trial had a treatment arm with pembrolizumab and olaparib in chemotherapy-experienced mCRPC patients, and observed an acceptable safety profile with indications of clinical activity in molecularly unselected disease with an 8.5% objective response and 14-month median OS 174. Olaparib is also showing promise in concert with the PD-L1 blocker durvalumab (Imfinzi) especially in DNA damage repair deficient mCRPC 175, in line with the synthetic lethal activity of PARP-inhibitors in the clinic.

Rucaparib demonstrated tolerable safety and encouraging activity in combination with PD-1 blockade in the CheckMate 9KD trial, where objective responses were 10.3% and 15.4% for post-chemotherapy and naïve cohorts, respectively, and OS was 13.9 and 20.2 months 158. Responses were greater in patients with homologous repair deficiencies, especially in *BRCA1/2*-mutated subpopulations where 33.3% objective responses were observed in each cohort. When the combination advanced to the KEYLYNK-10 Phase III trial against enzalutamide and abiraterone, however, primary endpoints were not met and the trial was stopped 176. Despite this setback and limitations in study design that disregard ICI monotherapy, the results presented outside of the trial favor further investigation into the PARP-inhibitor/ICI combination regimen, especially where patients are expected to harbor mutations that increase neoantigen load (see NCT04336943).

Although other targeted agents have not performed well as monotherapies, a number of candidates are being tested for potential benefit when combined with checkpoint blockers in PCa. Targets range from members of dysregulated pathways such as AKT (NCT03673787), receptor tyrosine kinases (RTKs) (NCT04848337), and cyclin-dependent kinases (CDKs) (NCT04751929), to epigenetic readers that support the function of transcriptional master regulators which in turn fuel oncogenic networks (NCT04471974) 177. The RTK inhibitor cabozantinib demonstrated manageable safety and minor clinical activity when combined with atezolizumab in the mCRPC cohort of the ongoing Phase Ib COSMIC-021 trial, with a 32% ORR and a confirmed PSA response in 50% of evaluable patients 178. This outcome laid the foundation for the Phase III CONTACT-02 trial, where mCRPC patients failing a single NHT were randomized to receive cabozantinib and atezolizumab or a second cycle of NHT (NCT04446117).

Preliminary results from the trial have shown a significant increase in median PFS with 6.3 vs 4.2 months and a median OS of 16.7 vs 14.6 months in the control group, but the study has sparked debate due to the modest values and questionable study design 179, 180. The primary concern is the inclusion of a control regimen that delivers a second NHT, which is not considered the best standard of care treatment for the study group when taxane chemotherapy exists as a more viable treatment option 180. With a median follow-up of 12 months, the reported OS is immature and unfit for making justified claims about the benefits of treatment. The study may yet show promise, but the final report will need to present more robust data for it to have significant implications.

## Enhancing the effectiveness of checkpoint inhibition by novel combination strategies in pre-clinical models of PCa

As reviewed above, since clinical trials for ICI monotherapy in PCa have largely been unsuccessful so far, current emphasis is on combination therapies with agents that are already in the clinic. As we wait for the decisive outcome of these trials, it is important to explore additional combinatorial approaches based on the information that has accumulated on the biology of PCa over the years, which has to be supported by robust findings in preclinical models. This has in fact been ongoing for some time and below is an overview of such preclinical studies that may find translational applications in the future (**Table 4**).

Similar to what has been observed in humans, combining anti-CTLA-4 and anti-PD-1 ICIs has resulted in only modest efficacy in mouse PCa models 181. However, when immune checkpoint blockade was combined with MDSC-targeted therapy using multikinase inhibitors, such as cabozantinib and phosphoinositide 3-kinase (PI3K)/mTOR dual inhibitor BEZ235, there were robust synergistic tumor burden decreases in both primary and metastatic CRPC in murine models 181. In another study involving MDSCs, lymphocyte-specific protein tyrosine kinase (LCK), a key protein for T cell activation, was shown to be nitrated and rendered inactive by reactive nitrogen species (RNS) generated by MDSCs in ICI resistant PCa tumors 182. In a mouse model of CRPC, where Pten, p53, and Smad4 are specifically deleted in the prostate, CRPC exhibited resistance to anti-PD-1 and CTLA-4 agents. However, the effectiveness of ICI infusion was significantly enhanced when combined with uric acid which acts as an RNS neutralizing agent 182. Taken together, these findings suggest that combining immune checkpoint blockade with MDSC-targeted therapies may be a viable treatment option for mCRPC 181, 182.

More recently, Peng *et al.* introduced a novel therapeutic strategy that targets the prostaglandin E2 receptor EP4 (PTGER4) that is found in various immune cells 183. This strategy involves using a recently identified EP4 antagonist called YY001, effectively reversing the immunosuppressive characteristics of MDSCs while simultaneously boosting the infiltration and activity of CD8^+^ T cells. Combination of YY001 with anti-PD-1 therapy proved to be highly effective in inhibiting tumor progression. This combination led to long-term survival and the development of enduring immunologic memory. These findings suggest a transformation of the TME from an immunologically cold state, where immune response is limited, to an immunologically hot state, where the immune system actively targets and eliminates cancer cells 183.

Pten-deficient PCa mouse models typically exhibit resistance to anti-PD-1 immunotherapy. However, recent research suggests that intermittent administration of the PI3Kα/β/δ inhibitor BAY1082439, as opposed to continuous daily dosing, can effectively mitigate development of resistance 184. This treatment regimen fosters increased infiltration of CD8^+^ T cells into the TME and augments the antitumor immune response, effectively transforming previously non-responsive cold tumors into "T-cell-inflamed" tumors. These findings represent a promising approach to enhance the efficacy of immunotherapy for Pten-null PCa 184. Consistent with these findings, the combination of ADT degarelix and PI3K inhibitor copanlisib showed a partial antitumor response in a murine PCa model with Pten/p53 deficiency 185. This response was achieved by increasing the frequency of activated TAMs in the TME. However, the addition of anti-PD-1 to copanlisib did not lead to a higher overall response rate. Nevertheless, when mice were treated with degarelix + copanlisib + anti-PD-1 combination therapy, there was a 60% increase in ORR within 28 days compared to untreated controls 185.

Implicating another central signaling pathway in the ICI response, we recently discovered that genetic deletion or small molecule inhibition of one of the canonical unfolded protein response pathways, IRE1α-XBP1s, increased response to PD-1 therapy in syngeneic mouse models 186. CRISPR/Cas9-mediated deletion of IRE1α or treatment with its small molecule inhibitor MKC8866 (ORIN1001), which is currently in clinical trials, reprogrammed the TME, reversed immunosuppression, increased NK and CD8^+^ T-cell infiltration, augmented interferon responses, and enhanced the efficacy of anti-PD-1 therapy in various PCa syngeneic mouse models 186. Furthermore, in the same study, we discovered a novel TAM gene signature that is associated with poor PCa survival that is significantly decreased by the combination of MKC8866 and anti-PD-1 therapy. These findings suggest that IRE1α inhibition could potentiate the effectiveness of anti-PD-1 immunotherapy in PCa. Further work is required to evaluate the translational potential of these findings.

In another study, anti-PD-1 immunotherapy combined with patient-derived prostate-specific microbe CP1 injection, there was notable improvement in survival rates and a reduction in tumor size in orthotopic models of MYC- and PTEN-mutant PCa syngeneic models 187. CP1 injection enhanced the immunogenic cell death of cancer cells, boosted T cell cytotoxicity, and promoted the infiltration of activated CD8^+^ T cells as well as other cell types such as NK cells, M1 macrophages, and mature dendritic cells into the tumor. Consistently, durable antitumor effects and extended survival were achieved and the potential for a cure was observed in a syngeneic PCa model when anti-B7-H3 inhibitor was combined with enzalutamide and the blockade of PD-L1 or CTLA-4 188.

In tumors with limited T-cell infiltration and poor response to radiation therapy, combining an agonistic anti-CD40 monoclonal antibody (mAb) led to a reprogramming of the TME 189. This reprogramming involved increased IFN-γ signaling, activation of Th-1 pathways, and higher infiltration of CD8^+^ T cells into the TME. As a result, the combination therapy showed better tumor control compared to using radiation and anti-PD-1 alone Moreover, this regimen increased the presence of Tregs and engaged the CTLA-4 axis within the TME. When anti-CTLA-4 antibody was administered alongside radiation therapy and anti-CD40 mAb therapy, it overcame Treg-mediated immune suppression, resulting in a higher ratio of cytotoxic T cells, tumor rejection, and the development of long-term immunity 189. Consistently, combining Radium-223 and degarelix with ICIs targeting PD-1 and CTLA-4 demonstrated superior efficacy compared to using each treatment alone in the Myc-CaP bone-tumor bearing mouse model 190.

The effectiveness of checkpoint therapy was also augmented by combining chemotherapy drugs with ICIs. For example, docetaxel treatment activated the cGAS/STING pathway in PCa, leading to the induction of IFN signaling and subsequent infiltration of lymphocytes into the tumor 153. In a mouse model, a chemohormonal therapy based on docetaxel facilitated the intratumoral infiltration of T cells and sensitized the mouse tumors to anti-PD-1 blockade. To evaluate the clinical significance of these findings, a retrospective analysis was conducted on 30 metastatic CRPC patients. The results showed that combining docetaxel with anti-PD-1 antibody tislelizumab led to improved PSA progression-free survival for patients with a ≥25% PSA reduction compared to using tislelizumab alone 153.

Endogenous tumor-specific tissue-resident memory T (TRM) cells have emerged as a focal point in cancer immunotherapy research 191. In a murine model of PCa, a novel dual therapy approach combining primary tumor destruction using irreversible electroporation, followed by anti-CTLA-4 treatment not only confirmed the establishment of TRM cells but also demonstrated their pivotal role in conferring protection against subsequent tumor challenges. Building upon this success, a triple-therapy strategy that included anti-PD-1 antibodies showed remarkable efficacy in cases that had initially shown resistance to treatment 192. In another study, in the TRAMP-C2 model, the combination therapy of cryoablation and CTLA-4 blockade displayed remarkable synergy, leading to the rejection of a second tumor challenge 193. Tumors exhibited increased infiltration of CD8^+^ T cells at the challenge sites and the combination therapy group showed a higher ratio of effector T cells to Treg cells. Furthermore, an independent study demonstrated that the combination of cryoablation, degarelix and CTLA-4 blockade resulted in a synergistic effect, leading to a notable delay in the growth of distant tumors and a reduction in the mortality rate 194.

In addition to these findings, combination of anti-CD73 antibodies with anti-PD-1 or anti-CTLA-4 treatment resulted in a substantial enhancement of antitumor activity in the RM-1 syngeneic mouse PCa model 195. In another study, the addition of anti-RANKL (receptor activator of nuclear factor kappa beta) to the combination therapy of anti-PD-1 and anti-CTLA-4 led to enhanced anti-tumor responses, regardless of the ability of anti-CTLA-4 isotype to engage activating Fc receptors 196. Both concurrent and delayed RANKL blockade proved to be highly effective. An early assessment during treatment showed that this triple combination therapy, when compared to the dual combination of anti-PD-1 and anti-CTLA-4, further increased the proportion of tumor-infiltrating CD4^+^ and CD8^+^ T cells capable of producing both IFN-γ and TNFα 196.

In a number of different studies, small molecule inhibitors were combined with ICIs in PCa pre-clinical models. For instance, the efficacy of PD-L1 blockade in the TRAMP-C2 model was significantly improved by using A485, a small molecule inhibitor that targets p300/CBP transcriptional coregulators 197. A485 effectively blocked both the intrinsic and IFN-γ-induced PD-L1 expression. As a result, the combination of the inhibitor with PD-L1 blockade had a significantly enhanced efficacy in this model 197. In another study, combination therapy involving ATR inhibitor (ATRi) BAY1895344 and anti-PD-L1 demonstrated greater inhibition and survival of tumor bearing mice compared to using either of the individual agents alone in RM-1-BM mouse PCa model 198. The combined administration of ATRi and anti-PD-L1 therapy led to strong activation of the innate immune system and a synergistic therapeutic response that was T-cell dependent.

In another study, targeting BET bromodomains with the small molecule inhibitor JQ1 led to a reduction in PD-L1 expression and inhibited tumor progression in PCa models 199. This effect was associated with an increase in MHC class I expression and immunogenicity of the tumor cells. Moreover, in the Myc-CaP syngeneic PCa model, combining JQ1 treatment with anti-CTLA-4 immunotherapy resulted in an additive effect, leading to an increased CD8/Treg ratio, which is beneficial for antitumor immune responses 199.

Morel *et al.* used the small molecule inhibitor EPZ6438 that targets enhancer of zeste homolog 2 (EZH2) of the Polycomb repressive complex 2 (PRC2) to activate a stress response involving double-stranded RNA-STING-Interferon-Stimulated Genes (ISG) pathway 200. This activation leads to the upregulation of genes related to antigen presentation, Th1 chemokine signaling, and interferon response, including PD-L1. As a result of EZH2 inhibition, there was a significant increase in the infiltration of activated CD8^+^ T cells and M1 TAMs within the tumor 200. This reversal of resistance to PD-1 checkpoint inhibition demonstrated the potential of EZH2 inhibition as a promising therapeutic strategy to enhance effectiveness of ICI therapy in PCa.

## Discussion and future perspectives

ICIs have revolutionized the treatment landscape in some cancer types. However, they have so far failed to show efficacy in Phase III trials on PCa. This has been linked to the cold immunophenotype of the prostate TME, characterized by a quantitative deficiency in cytotoxic T cells and a plethora of immunosuppressive mechanisms that curb the antitumor response. A low mutational burden and scarcity of neoantigen formation that is necessary to mobilize the adaptive immune system limit antigen recognition on PCa cells. This in turn, does not allow “releasing the brakes on the immune system”, which is the goal of checkpoint blockers. Can this be reversed in some way in the case of PCa?

The current preclinical and clinical research findings suggest that understanding the immunophenotype of PCa mechanistically may be critical to improve the efficacy of ICIs for PCa. Whereas some studies have suggested that blockade of different checkpoints produces unique effects in the immune compartment 123, 124, dual checkpoint inhibition using monospecific antibodies or bispecific constructs has demonstrated clinical activity 125, 126, 133, 141, 142. The compensatory upregulation of other checkpoints under monotherapy further supports this strategy 116, and the emergence of less well studied checkpoints to date suggests that new targets may be available when other options fail 119, 129, 131. A significant challenge is that our knowledge on each checkpoint from a functional point of view is yet very limited, and some present with contradictory functions in the tumor landscape 127. An important task for the future is to identify as to which targets are most actionable and which combination therapies would prove most effective. As a related but different strategy, therapeutic benefit may be achieved by blocking checkpoints and simultaneously depleting immunosuppressive cytokines from the TME 149, but evidence suggests that one can alternatively aim to circumvent immunosuppressive stimuli by supercharging costimulatory markers with TAA-CTL bridging bispecifics and complement with checkpoint targeting antibodies 140.

Given the low mutational burden of PCa, checkpoint inhibition may still not achieve its full potential until the immune system is primed to recognize the tumor as harmful, which involves a complex interplay between innate and adaptive immune compartments. Anticancer vaccines are the most straightforward option, but few vaccines have proven effective by themselves, and ideal antigen selection is difficult. Some studies have demonstrated a potential for ICIs and vaccines to synergize 148, 151, but the clinical benefit of this approach in PCa is not known. Current standard of care treatments have shown immunomodulatory and proinflammatory activities which has suggested that they could be combined with ICIs in PCa therapy 153, 159, 161, 170. However, this raises an important issue regarding the timing of checkpoint combinations in a patient's treatment history; would a patient benefit more from combination regimens in the early phases of PCa or further down the course of the disease?

Perhaps the main issue in the ICI efficacy for PCa relates to the subject of patient selection in clinical trials. It is clear from previous trials that a select subset of patients respond more favorably to checkpoint mono- and combination therapies, but the full characteristics of these patient subsets are not known 22, 23, 30, 125, 173. Although biomarkers such as PD-1 expression, CD8^+^ infiltration, TMB, and mismatch repair deficiency are repeatedly associated with better outcomes in response to different ICIs, attempts to stratify patients based on these characteristics have had limited success. Emerging biomarkers - CD73, VISTA, NK-cell markers and more - provide alternative routes to making informed decisions in future trials. Furthermore, a nested study design that takes multiple biomarkers into account may be required to optimize their utility in predicting treatment responses for PCa patients. The observation that blockade of different checkpoints enforces different changes in the immune landscape 123 suggests that biomarkers should not only inform a binary decision on ICI eligibility, but which checkpoints need to be co-targeted and how they can synergize with targeted therapies that are already showing promise in such combination regimens (e.g. 175, 178, 199). This again requires deeper knowledge into the mechanism(s) underlying each checkpoint in the PCa TME and the concurrent impact of actionable targets in cancer cells.

Given that MKC8866 is currently in clinical trials (NCT03950570) for advanced cancer patients, our findings indicate that new clinical trials could be developed for PCa, incorporating anti-PD-1 immunotherapy with IRE1α inhibition. Beyond therapeutic potential, the TAM gene signature we identified demonstrates significant prognostic value in prostate cancer patients and is diminished by the combination of MKC8866 and anti-PD-1 therapy. This suggests that the TAM gene signature could be useful in predicting disease progression and tailoring treatment strategies. For instance, it may assist in stratifying prostate cancer patients for anti-PD-1 immunotherapy. Further research is needed to explore these possibilities.

In summary, current evidence suggests that ICIs hold an untapped potential in PCa; however, the cold immunophenotype poses a significant challenge to harnessing it. Chemotherapeutics and targeted therapies have demonstrated the potential to modify this phenotype for clinical benefit. Careful biomarker-based patient selection with informed combination regimens is therefore emerging as a means to optimizing ICI activity in the clinic. More knowledge is needed as to which biomarkers are suitable guides and actionable target nodes, but the progress thus far suggests that the future may present new possibilities to transform the cold and barren PCa TME to one that responds to checkpoint inhibition and other immunotherapies.

## Figures and Tables

**Table 1:** Ongoing or recently completed clinical trials with ICI combinations in PCa. Target group is specific for mCRPC unless otherwise specified.

Clinical trial ID	Common name	Combination^1^	Phase	Status	Enrollment^2^/study type	Start	Est. study completion
NCT02985957	CheckMate 650	Ipilimumab (CTLA-4) + Nivolumab (PD-1)	2	Active, not recruiting	351 Randomized, Open Label	March, 2017	January, 2025
NCT05708950*	VISTA-101	KVA12123 (VISTA) + Pembrolizumab (PD-1)	1/2	Recruiting	314 Randomized, Open Label	March, 2023	December, 2024
NCT02740985*	REFMAL 435	AZD4635 (A2AR) + Durvalumab (PD-L1)	1	Completed	313 (45) Non-randomized, Open Label	June, 2016	March, 2023
NCT04089553	-	AZD4635 (A2AR) + Durvalumab (PD-L1); AZD4635 + Oleclumab (CD73)	2	Completed	59 Randomized, Open Label	August, 2019	April, 2023
NCT02788773	-	Durvalumab (PD-L1) + Tremelimumab (CTLA-4)	2	Active, not recruiting	52 (39) Randomized, Open Label	August, 2016	June, 2024
NCT02465060*	NCI MATCH Molecular Analysis for Therapy Choice	Nivolumab (PD-1) + Relatlimab (LAG-3)	2	Active, not recruiting	6452 Non-randomized, Open Label Genetic screening based (main purpose)	August 2015	December, 2025
NCT03333616**	-	Ipilimumab (CTLA-4) + Nivolumab (PD-1)	2	Active, not recruiting	100 (5) Single Group, Open Label	December, 2017	May, 2025
NCT03651271*	AMADEUS	Ipilimumab (CTLA-4) + Nivolumab (PD-1)	2	Completed	100 Non-randomized, Open Label Stratified by CD8^+^ density	October, 2018	June, 2023
NCT04717154	INSPIRE	Ipilimumab (CTLA-4) + Nivolumab (PD-1)	2	Active, not recruiting	69 Single Group, Open Label Patients with immunogenic signature by sequencing	January, 2021	February, 2026
NCT03061539	NEPTUNES	Ipilimumab (CTLA-4) + Nivolumab (PD-1)	2	Active, not recruiting	380 Non-randomized, Open Label Patients with immunogenic signature by IHC and sequencing	February, 2018	June, 2027
NCT03454451*	-	CPI-006 (CD73) + Ciforadenant (A2AR); CPI-006 (CD73) + Pembrolizumab (PD-1)	1/1b	Completed	117 Randomized, Open Label	April 2018	February, 2023
NCT04485013* PCa is currently reserved as keyword for the study, but not yet assigned to treatment arms.	-	Pembrolizumab (PD-1) + TTX-080 (HLA-G)	1a/1b	Active, not recruiting	240 Non-randomized, Open Label	July, 2020	June, 2024
NCT02861573 Arms G and H: mCRPC and treatment-emergent neuroendocrine mCRPC.	KEYNOTE 365	Pembrolizumab (PD-1) + Vibostolimab (TIGIT)	1b/2	Recruiting	1200 Non-randomized, Open Label	November, 2016	October, 2027

A2AR = adenosine A2A receptor, CD73 = cluster of differentiation 73, CTLA-4 = cytotoxic T-lymphocyte-associated protein 4, HLA-G = human leukocyte antigen-G, LAG-3 = lymphocyte activation gene-3, PD-1 = programmed cell death protein 1, PD-L1 = programmed death-ligand 1, TIGIT = T cell immunoreceptor with immunoglobulin and ITIM domain, VISTA = V-domain Ig suppressor of T cell activation. ^1^Combination may be one of several treatment arms in a larger study. Parentheses indicate the targeted checkpoint. ^2^Parentheses indicate the number of PCa patients included in a treatment arm for a multicancer study, or the number of patients receiving the specified treatment in a PCa-specific study with multiple arms, where this information is available. *Advanced solid malignancies and metastatic cancers (including but not exclusive to mCRPC). **Rare genitourinary tumors (includes rare and aggressive PCa subtypes).

**Table 2:** Ongoing or recently completed Phase II clinical trials with vaccination in combination with checkpoint inhibition or bifunctional checkpoint targeting agents that have previously been described. Target group is specific for mCRPC unless otherwise specified.

Clinical trial ID	Common name	Combination^1^	Phase	Status	Enrollment/ study type	Start	Est. study completion
NCT03315871 BCR PCa	-	PROSTVAC-V/F (viral prime-boost vaccine presenting PSA and TRICOM) + M7824 (PD-L1, TGF-ꞵ trap) + CV301 (viral prime-boost vaccine presenting CEA, MUC-1, and TRICOM)	2	Active, not recruiting	40 Non-randomized, Open Label	March, 2018	January, 2025
NCT03493945*	QuEST1	BN-Brachyury (viral prime-boost vaccine presenting brachyury transcription factor and TRICOM) + M7824 (PD-L1, TGF-ꞵ trap); BN-Brachyury + M7824 + N-803 (IL-15/IL-15R alpha superagonist complex); BN-Brachyury + M7824 + N-803 + Epacadostat (IDO1 inh.)	1/2	Active, not recruiting	53 Randomized, Open Label	May, 2018	December, 2024
NCT03600350 Non-metastatic BCR PCa	-	pTVG-HP (plasmid DNA vaccine encoding PAP) + Nivolumab (PD-1) + GM-CSF (APC growth factor)	2	Active, not recruiting	19 Single Group, Open Label	September, 2018	December, 2027
NCT02933255 mCRPC and localized advanced PCa	-	PROSTVAC-V/F (viral prime-boost vaccine presenting PSA and TRICOM) + Nivolumab (PD-1)	1/2	Completed	24 Non-randomized, Open Label	April, 2018	December, 2023
NCT04989946 Newly diagnosed, high risk PCa	-	Degarelix (GnRH antagonist) + pTVG-AR (plasmid DNA vaccine encoding AR ligand-binding domain) + Nivolumab (PD-1)	1/2	Recruiting	60 Randomized, Open Label	December, 2021	December, 2028
NCT02499835	-	pTVG-HP (plasmid DNA vaccine encoding PAP) + Pembrolizumab (PD-1)	1/2	Completed	66 Randomized, Open Label	July, 2015	July, 2023
NCT04090528	-	pTVG-HP (plasmid DNA vaccine encoding PAP) + Pembrolizumab (PD-1); pTVG-HP + pTVG-AR (plasmid DNA vaccine encoding AR ligand-binding domain) + Pembrolizumab	2	Active, not recruiting	60 Randomized, Open Label	October, 2019	October, 2026

AR = androgen receptor, BCR PCa = Biochemically recurrent prostate cancer, GnRH = gonadotropin-releasing hormone, HOXB13 = homeobox B13, IDO1 = indoleamine 2,3-dioxygenase 1, IL-15/IL-15R = interleukin-15/IL-15 receptor, KLK2/3 = kallikrein related peptidase 2/3, M7824 = bintrafusp alfa (anti-PD-L1 + TGF-ꞵ receptor II ligand binding domain), MMAE = monomethyl auristatin E, NK3 homeobox 1, PAP = prostatic acid phosphatase, RNA-LPX = RNA lipoplex, TRICOM = triad of costimulatory molecules (B7.1, intercellular adhesion molecule 1 (ICAM-1), and lymphocyte function-associated antigen 3 (LFA-3)), PD-1 = programmed cell death protein 1, PD-L1 = programmed death-ligand 1. ^1^Combination may be one of several treatment arms in a larger study. Parentheses indicate the targeted checkpoint or the properties of the treatment. *Advanced solid malignancies and metastatic cancers (including but not exclusive to mCRPC).

**Table 3:** Ongoing or recently completed Phase II and Phase III clinical trials with dual checkpoint inhibition in combination with targeted therapy or standard of care treatment in PCa. Target group is specific for mCRPC unless otherwise specified.

Clinical trial ID	Common name	Combination^1^	Phase	Status	Enrollment/study type	Start	Est. study completion
NCT05169684	-	BMS-986218 (CTLA-4) + Nivolumab (PD-1) + Docetaxel (microtubule inh.)	2	Completed	10 Randomized, Open Label	February, 2022	December, 2023
NCT03866382**	ICONIC	Cabozantinib (tyrosine kinase inh.) + Ipilimumab (CTLA-4) + Nivolumab (PD-1)	2	Recruiting	314 Single Group, Open Label	April, 2019	February, 2025
NCT05150236	EVOLUTION (ANZUP2001)	177Lu-PSMA-617 (radioligand) + Ipilimumab (CTLA-4) + Nivolumab (PD-1)	2	Active, not recruiting	93 Randomized, Open Label	April, 2022	December, 2024
NCT05655715	CheckPRO	SBRT + Ipilimumab (CTLA-4) + Nivolumab (PD-1)	2	Recruiting	90 Randomized, Open Label	November, 2019	January, 2025
NCT04709276 Neuroendocrine or aggressive variant PCa	CHAMP	Cabazitaxel (microtubule inh.) + Carboplatin (alkylating) + Ipilimumab (CTLA-4) + Nivolumab (PD-1)	2	Active, not recruiting	43 Single Group, Open Label	June, 2021	June, 2027
NCT03879122 Metastatic hormone-sensitive PCa	PROSTRATEGY	ADT + Docetaxel (microtubule inh.) + Ipilimumab (CTLA-4) + Nivolumab (PD-1)	2/3	Active, not recruiting	135 Randomized controlled, Open Label	February, 2019	December, 2024
NCT04169841*	GUIDE2REPAIR	Durvalumab (PD-L1) + Olaparib (PARP inh.) Tremelimumab (CTLA-4)	2	Active, not recruiting	270 Single Group, Open Label Molecular screening for mutations in homologous repair genes	February, 2020	August, 2027
NCT03518606*	MOVIE	Durvalumab (PD-L1) + Tremelimumab (CTLA-4) + Vinorelbine (microtubule inh.)	1/2	Active, not recruiting	126 Non-randomized, Open Label	June, 2018	December, 2024

^177^Lu-PSMA-617 = Lutetium radionuclide conjugated with a prostate specific membrane antigen (PSMA) targeting ligand, ADT = androgen deprivation therapy, CTLA-4 = cytotoxic T-lymphocyte-associated protein 4, PARP = poly ADP-ribose polymerase, PD-1 = programmed cell death protein 1, PD-L1 = programmed death-ligand 1, SBRT = stereotactic body radiation therapy. ^1^Combination may be one of several treatment arms in a larger study. Parentheses indicate the targeted checkpoint or the properties of the treatment. *Advanced solid malignancies and metastatic cancers (including but not exclusive to mCRPC). **Rare genitourinary tumors (includes rare and aggressive PCa subtypes).

**Table 4:** ICI combination treatment findings in PCa pre-clinical models.

Combination	Pre-clinical PCa model	Reference
Cabozantinib + CTLA-4 + PD-1 or BEZ235 + CTLA-4 + PD-1	*PB-Cre*^+^; *Pten^L^*^/*L*^ *p53^L^*^/*L*^ *Smad4^L^*^/*L*^ *mTmG^L^*^/+^ *LSL-LUC^L^*^/+^ (CPPSML) transgenic model	181
Uric acid + CTLA-4 + PD-1	*PB-Cre^+^; Pten^L/L^ p53^L/L^ Smad4^L/L^* (Pten/p53/Smad4-deficient) transgenic model	182
YY001 + PD-1	RM-1 cells subcutaneous and orthotopic syngeneic model MSK-PCa2 patient derived organoid tumor injected to humanized CD34^+^ mouse model	183
BAY1082439 + PD-1	*Pb-Cre^+^Pten^L/ L^(Pten-null) transgenic model*	184
Degarelix + copanlisib + PD-1	*Pb-Cre; PTEN^L/L^ Trp53^L/L^* (PTEN/p53-deficient) transgenic model	185
MKC8866 + PD-1	Myc-CaP WT or PTEN CRISPR knock-out cells or RM-1 cells subcutaneous syngeneic model	186
CP1 + PD-1	Myc-CaP WT or PTEN CRISPR knock-out cells orthotopic syngeneic model	187
Enzalutamide + anti-B7-H3 + PDL-1 or Enzalutamide + anti-B7-H3 + CTLA-4	DX1 cells subcutaneous syngeneic model	188
Radiotherapy + anti-CD40 + CTLA-4	TRAMP-C1 and DVL3 cells subcutaneous syngeneic model	189
Radium-223 + Degarelix + CTLA-4 + PD-1	Myc-CaP cells injected in the femur syngeneic model	190
Bicalutamide + docetaxel + PD-1	RM-1 cells subcutaneous syngeneic model	153
Irreversible electroporation + CTLA-4 + PD-1	TRAMP-C2 cells subcutaneous syngeneic model	192
Cryoablation + CTLA-4	TRAMP-C2 cells subcutaneous syngeneic model	193
Cryoablation + CTLA-4	Myc-CaP subcutaneous syngeneic model	194
anti-CD73 + CTLA-4 or anti-CD73 + PD-1	RM-1 cells subcutaneous syngeneic model	195
anti-RANKL + PD-1 + CTLA-4	TRAMP-C1 cells subcutaneous syngeneic model	196
A485 + PD-L1	TRAMP-C2-Ras cells subcutaneous syngeneic model	197
BAY1895344 + PD-L1	RM-1-BM cells subcutaneous syngeneic model	198
JQ1 + CTLA-4	Myc-CaP subcutaneous syngeneic model	199
EPZ6438 + PD-1	B6-HiMYC transgenic model	200

CTLA-4 = cytotoxic T-lymphocyte-associated protein 4, PD-1 = programmed cell death protein 1, PD-L1 = programmed death-ligand 1, Cabozantinib: multi-kinase inhibitor, BEZ235: PI3K)/mTOR dual inhibitor, YY001: the prostaglandin E2 receptor EP4 antagonist, BAY1082439: PI3Kα/β/δ inhibitor, Degarelix: androgen deprivation therapy agent, copanlisib: PI3K inhibitor, anti-RANKL: inhibitor of receptor activator of nuclear factor kappa beta, A485: a small molecule inhibitor of p300/CBP, BAY1895344: Ataxia telangiectasia protein kinase (ATR) inhibitor, JQ1: BET inhibitor, EPZ6438: Enhancer of zeste homolog 2 (EZH2) inhibitor.

## Abbreviations

ADT: Androgen deprivation therapy
AR: Androgen receptor
bsAb: Bispecific antibody
CRPC: Castration resistant prostate cancer
CTL: Cytotoxic T-lymphocyte
CTLA-4: Cytotoxic T-lymphocyte associated protein 4
ETS: E26 transformation-specific family
HLA: Human leukocyte antigen
ICAM-1: Intercellular adhesion molecule 1
ICI: Immune checkpoint inhibitor
LAG-3: Lymphocyte activation gene-3
MDSC: Myeloid-derived suppressor cell
mCRPC: Metastatic castration resistant prostate cancer
ORR: Objective response rate
OS: Overall survival
PARP: Poly (ADP-ribose) polymerase
PCa: Prostate cancer
PD-1: Programmed cell death protein 1
PD-L1/L2: Programmed death-ligand 1/2
PFS: Progression-free survival
PI3K: Phosphoinositide 3-kinase
PRC1/-2: Polycomb repressive complex 1/-2
PSA: Prostate-specific antigen
PSMA: Prostate-specific membrane antigen
TAA: Tumor-associated antigen
TAM: Tumor associated macrophage
TGF-β: Transforming growth factor β
TIM-3: T-cell immunoglobulin and mucin domain 3
TMB: Tumor mutational burden
TME: Tumor microenvironment
Treg: Regulatory T cell
TSA: Tumor-specific antigen
VISTA: V-domain Ig suppressor of T cell activation
